# Clustering of the Adult Population According to Behavioural Health Risk Factors as the Focus of Community-Based Public Health Interventions in Poland

**DOI:** 10.3390/ijerph20054402

**Published:** 2023-03-01

**Authors:** Anna Poznańska, Katarzyna Lewtak, Bogdan Wojtyniak, Jakub Stokwiszewski, Bożena Moskalewicz

**Affiliations:** 1 Department of Population Health Monitoring and Analysis, National Institute of Public Health NIH—National Research Institute, 24 Chocimska Street, 00-791 Warsaw, Poland; 2 Department of Social Medicine and Public Health, Medical University of Warsaw, 3 Oczki Street, 02-007 Warsaw, Poland; 3 Department of Health Promotion and Chronic Diseases Prevention, National Institute of Public Health NIH—National Research Institute, 00-791 Warsaw, Poland

**Keywords:** behavioural health risk factors, cluster analysis, Poland, health programmes, prevention

## Abstract

Effective lifestyle health promotion interventions require the identification of groups sharing similar behavioural risk factors (BRF) and socio-demographic characteristics. This study aimed to identify these subgroups in the Polish population and check whether local authorities’ health programmes meet their needs. Population data came from a 2018 question survey on a random representative sample of 3000 inhabitants. Four groups were identified with the TwoStep cluster analysis method. One of them (“Multi-risk”) differed from the others and the general population by a high prevalence of numerous BRF: 59% [95% confidence interval: 56–63%] of its members smoke, 35% [32–38%] have alcohol problems, 79% [76–82%] indulge in unhealthy food, 64% [60–67%] do not practice recreational physical activity, and 73% [70–76%] are overweight. This group, with an average age of 50, was characterised by an excess of males (81% [79–84%]) and people with basic vocational education (53% [50–57%]). In 2018, only 40 out of all 228 health programmes in Poland addressed BRF in adults; only 20 referred to more than one habit. Moreover, access to these programmes was limited by formal criteria. There were no programmes dedicated to the reduction of BRF exclusively. The local governments focused on improving access to health services rather than on a pro-health change in individual behaviours.

## 1. Introduction

Over the past ten years, local authorities have increasingly prioritised the health and well-being of local communities. Their activities concern various aspects of social life, including organisation of medical care, ensuring a clean environment, stable and affordable housing, safety, preventing addictions, and many others. In many countries, public health services have been entrusted to local governments by acts of national parliaments (in force, e.g., in the Netherlands since 2008, in Norway since 2012, and in England since 2013) [1]. Their actions are to equalise the distribution of factors that directly or indirectly affect the health of individuals and communities. Also, in Poland, local authorities, by law, carry out public health tasks. They develop, implement, and finance health programmes (called health policy programmes)—sets of actions targeting specific problems in their communities. This paper discusses interventions in the area of health promotion and disease prevention proposed by Polish local authorities to reduce behavioural risk factors.

Such actions are crucial in Poland, where the percentage of deaths due to cardiovascular diseases is distinctly higher than the average in the EU (in 2017, 43% vs. 37%) [2]. According to the Global Burden of Disease Study 2019, in Poland the high percentage of total deaths is attributable to behavioural (thus modifiable) risk factors (44% vs. 37% in UE) [3]. These numbers do not acknowledge the burden connected with excessive body weight, considered a metabolic factor, that contributes to a further 14.2% of deaths (against 10.9% in UE). Moreover, health problems in Poland are concentrated on specific demographic and social groups. According to EUROSTAT [4], the difference between men’s and women’s life expectancy equals eight years and is one of the highest among EU countries. Differences in health status and life expectancy have been reported for inhabitants of large cities and small towns [5]. The prevalence of smoking or overweight/obesity varies with the level of education [6].

An effective health policy in this area must recognise how harmful habits are distributed in the population [7]. Moreover, behavioural risk factors tend to aggregate, which has important implications for preventive medicine and health promotion [8]. This co-occurrence and the concentration of risk factors in specific population subgroups have already been thoroughly documented in many countries [9,10]. These are the cases of associating excessive drinking with smoking [8,10,11] and the concentration of bad habits among people who are less educated [10,12] and with lower socio-economic status [10,11,13]. Some of these correlations are ambiguous, e.g., the relationship between the level of physical activity and smoking differs in various countries and for various social groups [14,15].

In Poland, such studies are undertaken rarely and usually on a local scale—regarding professionally active subpopulations or inhabitants of one region [16]. The exception is the Polish Multicentre National Population Health Examination Survey (WOBASZ) that has been conducted twice (2003–2004 and 2013–2014). The comparison of both editions of this survey suggests that despite changes over ten years (both favourable—regarding smoking, as well as unfavourable—more frequent obesity, reduction in physical activity among men), the percentage of Poles with a healthy or unhealthy lifestyle remained unchanged: 2% and 25%, respectively [17]. However, no attempt has been made to describe population groups characterised by multiple risk factors. Considering the mentioned needs and the gap in knowledge concerning the co-occurrence of behavioural risk factors in the population of adult Poles, we undertook the study presented in this paper.

The main objectives of the study were:

- Identification of the groups of adult individuals in Poland who share various features in terms of risk factors for health (behavioural, overweight, lack of vaccination and preventive medical examinations) and socio-demographic characteristics based on the results of a nationwide survey;
- Checking whether the most exposed people find support in the interventions taken by local authorities by reviewing all health programmes proposed for realisation in the year of the survey.

## 2. Materials and Methods

### 2.1. Survey

The questionnaire survey on the prevalence of health risk factors conducted in Autumn 2018 was based on a random sample of 3000 inhabitants of Poland aged 20 and above. The sample was drawn from the Universal Electronic System for Registration of the Population (PESEL). In order to ensure the intended number of subjects at the expected response rate of 50%, 6000 people were drawn; the interviews were conducted until the assumed sample of 3000 respondents was reached. The sampling scheme used included the population stratification according to the province of living and residence location class (in 6 categories: rural areas, towns with a population of up to 20,000, 20–100,000, 100–500,000, 500,000–1 million, and the largest city in the country with 1.8 million inhabitants—Warsaw) and two stages of drawing lots (first communes within the strata, then inhabitants of the selected communes in the gender and age proportions appropriate for the stratum). The obtained sample was representative for the national population in terms of sex, age, province of living, and the share of urban and rural residents.

Experienced interviewers conducted the survey using the computer-assisted personal interviewing (CAPI) method. The collected data regarded socio-demographic characteristics, height and body weight (in self-assessment), selected lifestyle-related health behaviours, use of medical care, and financial difficulties.

The results of the survey allow for the estimation of risk factor prevalence in the national population (after corrections for differences in sex and age structures between the final sample and the population). They also enable the identification of groups of individuals who share health behaviours and socio-demographic characteristics, i.e., involving people potentially in need of assistance in similar scope and form.

### 2.2. Statistical Analysis

In order to identify the groups mentioned above, cluster analysis was used, grouping individuals not risk factors. The TwoStep cluster analysis method was applied. It is often utilised in similar studies because it enables the simultaneous use of continuous and categorical variables and aids in determining the optimal number of clusters—their number does not necessarily need to be known a priori [11,13]. The following variables were used for cluster identification:

➢ Binary
  - Sex;
  - Marital status (in the following layout: married or cohabitant vs. single);
  - Living in rural (vs urban) areas;
  - Smoking (currently);
  - Problems with alcohol (affirmative answer for 3 questions: (a) Have you ever thought you were drinking too much alcohol? (b) Have people ever irritated or annoyed you with their comments regarding your alcohol-drinking habits? (c) Have you ever felt bad or felt guild because of drinking alcohol?);
  - Overweight (BMI ≥ 25);
  - Lack of recreational physical exercises (sport, gymnastics, jogging, cycling, etc.)—spending less than 10 min per week on physical activity resulting in at least raised respiratory or heart rate during the spring–summer and autumn seasons;
  - Unhealthy products in one’s diet (fast food meals; sweet, carbonated beverages; or sweets several times a week);
  - Too little vegetable/fruit intake in one’s diet (fewer than 5 portions a day);
  - Eating fish less frequently than once a week;
  - Lack of preventive medical examinations (diagnostic laboratory tests, cytology, mammography, colonoscopy) or vaccination in the last 3 years;
➢ Categorical
  - Education (in the following layout: primary, basic vocational, secondary, tertiary);
➢ Quantitative
  - Age (in years).

After identifying clusters, their characteristics were found within the scope of the above variables (percentage or median with a 95% confidence interval—95% CI presented in square brackets). Analogous values were also calculated for additional features (including financial difficulties—insufficient money to buy food, basic clothes, or paying monthly bills in the last year—and the need for medical consultation in the last year) not included directly in the clustering procedure due to their correlations with the used variables. They were used in the discussion of obtained results.

The chi-square and Kruskal–Wallis tests were applied for qualitative and quantitative variables, respectively, when comparing the characteristics determined for particular clusters. The statistical significance of observed differences was adjusted for multiple comparisons (Bonferroni correction). In order to eliminate the influence of differences in the age structure and education level between the compared groups on the prevalence of overweightness and obesity, direct standardisation of rates was applied; the national population served as the reference population.

In all statistical tests, the assumed significance level was 0.05. The analysis was conducted with the use of SPSS12.PL package.

### 2.3. Health Programmes

In the next stage of the study, the health programmes planned for implementation in the year of the survey (2018) were analysed to determine the extent to which they reflected the population’s needs in limiting behavioural risk factors.

The complete data come from the ProfiBaza information system [18], which stores information about public health interventions in Poland, including all health programmes submitted for assessment by the state Agency for Health Technology Assessment and Tariff System (AOTMiT). Under the provisions of law, the realisation and financing of each programme needs the sanction of the President of this institution.

## 3. Results

### 3.1. Cluster Analysis

The prevalence of main health risk factors in the studied group can be considered an estimate for the Polish population; differences in results after adjustment for the age structure do not exceed 0.5 percentage points. In Poland, 30% [29–32%] of the population smoke, 13% [12–15%] have drinking problems, 67% [65–68%] indulge in unhealthy products in their diet, the same percentage eat too few vegetables and fruit, 47% [45–48%] do not practice physical activity in their free time, 50% [48–51%] are overweight, and 44% [42–45%] do not undergo preventive medical examinations or vaccination (Table 1).

The analysed characteristics are not distributed evenly in the population; thus, four population clusters can be distinguished. These received the subjective names: 1—“The youngest” (covering 29% [27–31%] of the adults), 2—“Multi-risks” (26% [25–28%]), 3—“The oldest” (27% [25–28%], 4—“Healthy lifestyle” 18% [17–20%]. Relative to the general population, the main risk factors in cluster 1 are a high number of unhealthy products in the daily diet and no vaccination/preventive medical examinations. In cluster 2, all risk factors occur much more frequently than in the general population. Cluster 3 is characterised by a lack of physical activity and overweight/obesity. In cluster 4, all risk factors are significantly less common than in the general population. The description of the clusters is shown in Table 2.

The distributions of particular features and statistical significance of differences between the clusters are presented in Table 1 (variables used in the clustering procedure) and Table 3 (additional characteristics of identified clusters not directly used in the clustering process).

**Figure 1:**
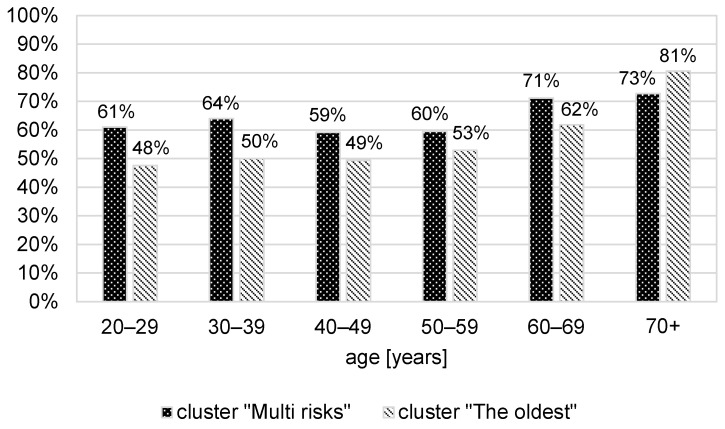
Percentage of people who do not participate in recreational physical activity in clusters “Multi risks” and “The oldest”, depending on their age.

**Figure 2:**
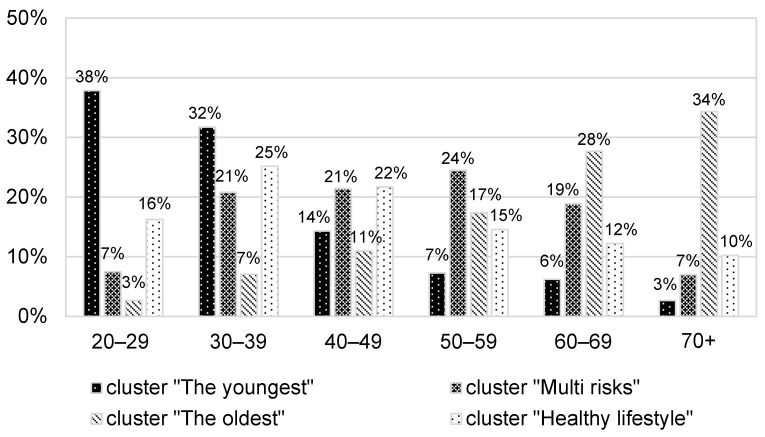
The age structure of identified clusters members.

The “Multi-risks” cluster unfavourably deviates from the other clusters in terms of the prevalence of behavioural risk factors; only the lack of recreational physical activity is as frequent (64%) as among “The oldest”. The latter group, however, consists of people on average 14 years older and, as indicated by the age-specific rates, more active up to the age of 69 (Figure 1). Equal percentages of inactive people result from a high excess of people over 70 years of age in this cluster. The age structure of members of all clusters is presented in Figure 2.

Extra body weight constitutes a severe problem in two clusters—it concerns 90% of “The oldest” and 73% of “Multi-risks” clusters; the percentage of obese people is 24% and 14%, respectively. In this case, age-specific coefficients among “The oldest” are much higher—after standardisation by age, the percentage of overweight people was 94% vs. 71%, whereas the obesity rate was 22% vs. 14%. The effects also do not originate from differences in the education structure—after standardisation by education level, the overweight rate is 93% vs. 75%. Among “The oldest”, the problem prevails more often, despite the clearly healthier diet (Table 1 and Table 3).

### 3.2. Review of Health Programmes

**Figure 3:**
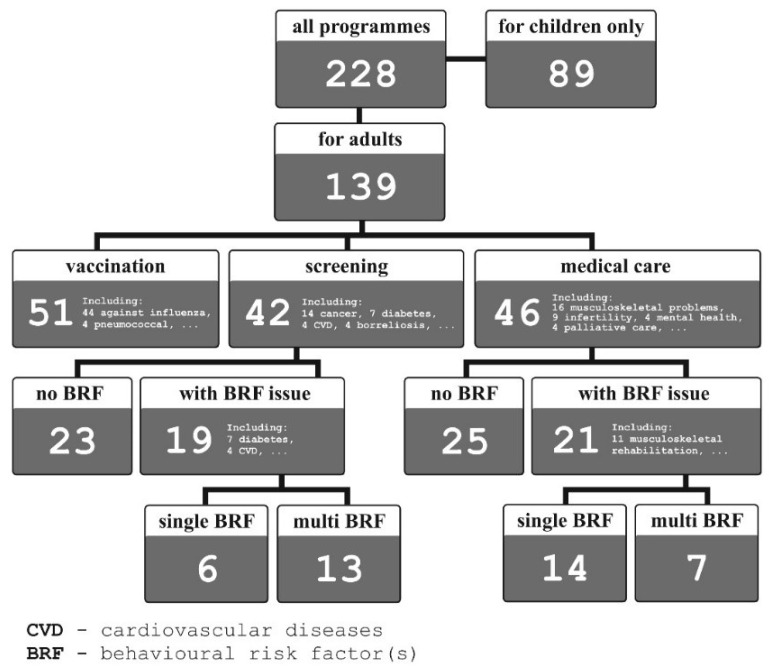
The number and nature of Polish health programmes submitted for assessment of AOTMiT in 2018.

In 2018, AOTMiT received 228 health programmes for assessment. Local governments had developed 97% of them for realisation in their administrative units. The Ministry of Health submitted the remaining seven programmes (3% of the total) for nationwide application. Their nature is summarised in Figure 3.

Almost 40% of the total were intended solely for children and adolescents. Adults were most often (in 51 out of 139 programmes) offered vaccination (optional in the country, the most often against influenza—Figure 3). In 80% of cases available to people over 60 years of age. As many as 46 programmes were devoted to the improvement of accessibility of healthcare for people with diagnosed health problems. Forty-two programmes offered participation in the screening examination.

Among health programmes aimed at adults, 40 (29%) dealt with the issue of behavioural risk factors, either in the context of healthcare or diagnostics. Half of the programmes in question acknowledged intervention in the scope of one behavioural factor (physical activity in 12 cases, nutrition in 5, smoking in 3), 11 programmes combined physical activity and nutrition, whereas 9 addressed three or more factors (Table 4).

All health programmes precisely specify the age range of recipients; 34% of programmes directed at adults involved people solely over the age of 60 or 65. However, there are no consistent, medically, and socially justified criteria for determining the age limits for the availability of these programmes, e.g., the difference in the eligibility age between particular osteoporosis prevention programmes is 10 years.

The aim of this study has not been to assess the substantive aspects of the presented health programmes (AOTMiT negatively reviewed 19% of programmes directed at adults, but formal shortcomings of the projects could also cause it). However, the presented data indicate that their authors neither consider the co-occurrence of risk factors nor the characteristics of groups with such unfavourable habits.

## 4. Discussion

This study identified four clusters in the Polish population, each of which shared the same health risk factors. Similarly to other countries, a group with a healthy lifestyle was found [9]. Age-related factors characterised the following two clusters: “The youngest”—the most physically active, overusing unhealthy food, and not interested in vaccination or preventive examinations; and “The oldest”—mostly women, avoiding smoking and alcohol, low physical activity, and generally overweight (90% overweight, 24% obese). Such phenomena as excessive consumption of fast-food meals by young people or low physical activity of older women are well known [11].

The most significant outcome is the identification of the “Multi-risks” cluster that combines most behavioural health risk factors. It seems that this group, constituting approximately one-fourth of the adult population, determines the high excess mortality rate of men in Poland. It mainly consists of males (81%), 59% smoke, 35% have alcohol problems, 83% eat too few vegetables and fruit, 79% indulge in unhealthy food, 64% are physically inactive, and 73% are overweight. The average age of its members is 50; more than half have basic vocational education. The existence of such a group has been reported in other countries [9,13]. It was also observed that subgroups with lower education engage in poor behaviours more often [7,10,11]. This “Multi-risks” group needs urgent intervention in the field of health promotion, also undertaken at local levels, aimed at lifestyle changes to help eliminate or limit several risk factors in one person.

Meanwhile, local authorities in Poland mainly focus on providing access to medical services—one-third of the health programmes directed at adults were devoted to treating or rehabilitating people with a diagnosed disease. In the scope of prevention, adult inhabitants were most often offered free vaccination (37% of programmes).

Other limitations in the availability of programmes result from the recipients’ age; 40% of programmes are intended for children and adolescents and 34% target adults over 60 or 65. Consequently, there is a shortage of programmes involving people at about 50 years of age who have multiple health risk factors but have not been diagnosed with one of the supported diseases—only 12 such programmes were available in 2018 (9% of these devoted to adults).

Local authorities do not implement health programmes aimed solely at reducing the prevalence of health risk factors. Although included in 29% of adult-oriented programmes, they were always combined with rehabilitation (thus intended for patients) or screening. Moreover, half of them regarded only one risk factor.

The effectiveness of multiple-risk interventions remains open. In general, considering the synergy of individual risk factors and the economic aspect of intervention or the lack of it, such actions have a more significant impact on public health than those targeted at single risk factors [9]. However, comparing the effectiveness of both strategies (simultaneous vs. sequentially delivered multiple health behaviour change interventions) can be inconclusive [19]. Moreover, a meta-analysis of 69 trials involving over 73,000 people revealed that interventions covering education and skill training aimed at many risk behaviours simultaneously, only result in changes concerning daily diet and physical activity, whereas the strategy of simultaneous reduction of smoking and other risk factors might be sub-optimal [20]. Regardless of the effectiveness of particular strategies of multiple risk interventions, even if a person manages to eliminate one risk factor, they may have no chance of receiving support for the successive elimination of further factors.

The efficiency of Polish health programmes is additionally affected by the fact that they do not differentiate the scope or methods regarding recipients’ sex or education level and neglect their culture of health (conditioned by age, education, and social status). People from the identified clusters differ in terms of lifestyle and attitude towards their own health—they represent diverse cultures of health. Over one-third of people in the “Multi-risks” cluster did not feel the need to receive medical help or even consultancy in the last year, and almost three-quarters did not undertake any preventive measures. Their physical activity is substantially lower than among “The youngest” (inactive 64% vs. 24%). This difference does not result only from their age. The percentage of inactive members of the “Multi-risk” cluster at 20–40 already exceeds 60% (Figure 1). On the other hand, both “The oldest” and “Healthy lifestyle” clusters comprise mainly females who do care for their health (diet and preventive actions). They clearly differ, however, in terms of age, education level, financial resources (frequency of financial difficulties), and also, possibly, the social support level (frequency of being in a long-term relationship) and opinion on socially accepted women’s behaviours (smoking and alcohol). Different problems in these groups require individualised solutions regarding the provided information and training of particular personal skills. Meanwhile, to increase the persuasive effect of communications in health promotion, effectively broadening recipients’ knowledge and their preparation for making medical decisions, it is recommended to apply culture-sensitive health communication adjusted to beneficiaries’ cultural backgrounds [21].

Thus, tailored interventions, even those conducted using computer-aided methods, are strongly recommended [22,23]. For instance, the acknowledgment of the occupational setting discussed concerning blue-collar workers [24] can be of great importance in Poland, where three-quartes of people in the “Multi-risks” cluster are of professionally active age (below 60) and over half have a basic vocational education.

It should be concluded that local governments’ activities in health promotion and disease prevention are insufficient to ensure control over risk factors for a national population of almost 38 million. There is a need for interventions at the central level, realised with the use of primary health care [25] and maybe also occupational medicine (the effectiveness of workplace-based policies is still under debate) [26]. Nevertheless, any party undertaking such actions, including local governments, should consider the existence of a group particularly affected by behavioural risk factors that need urgent and comprehensive help [11]. These people should be the target of appropriate interventions for this reason, not as residents of a certain age or patients needing treatment or rehabilitation for a specific disease. This study is aimed at identifying and describing this group.

The limitation of the study that should be discussed is the age of the subjects. The analysis of the prevalence of behavioural risk factors, the identification of clusters, and the review of available health programmes concern people aged 20 years or older. However, it has already been proven that many harmful health behaviours start at a younger age. Adult smoking begins in adolescence [27] and nutrition in childhood influences the risk of later obesity [28]. The Health Behaviour in School-aged Children (HBSC) study results show that among 15 year olds in Poland in 2018, 12% regularly smoked (including 5% daily), 26% ate sweets every day, and only 27% met the WHO recommendations for moderate-to-vigorous physical activity [29]. The analysis of health programmes addressed to children and adolescents is purposeful and planned to be carried out in the future.

The timing of the question survey (2018), i.e., before the outbreak of the COVID-19 pandemic, may also be questionable. However, it turned out that in the following years, the number of health programmes decreased significantly [18]. This tendency was evident during the COVID-19 pandemic. In 2019, 195 programmes were submitted for assessment, in 2020 it was 97, whereas in 2021there were only 80. At the same time, the need for aid increased. In many countries, the lockdown unfavourably affected the population’s health behaviours. The prevalence of overweightness and obesity increased due to limited physical activity and changes in dietary habits (eating more frequently and snacking) [30,31,32,33]. In Poland, there is a visible aggravation of previously practised unfavourable habits—over 45% of smokers did it more frequently during the lockdown and a stronger tendency to drink more was found among alcohol addicts. Similarly, older, so in general, heavier people were more likely to gain weight, whereas those underweight tended to lose it further [34]. These results confirm that the survey has not lost relevance and suggest that the population grouping according to risk factors could become even stronger. Thus, tailored interventions aimed at reducing multiple risk factors will be increasingly needed to prevent further consolidation of risk factors in certain social groups that would exacerbate the previously observed health inequalities [33].

## 5. Conclusions

Among inhabitants of Poland, one can distinguish four population groups that differ in terms of the prevalence of behavioural health-related risk factors and socio-economic situation. Similarly to other countries, a “Multi-risks” cluster was identified. It constitutes approximately one-quarter of the adult population and differs from other groups and the general population, with a high prevalence of numerous lifestyle-related health risk factors.

The existence of the said group, comprising mostly men, can be related to the phenomenon of excess male mortality and the big difference (8 years) in the life expectancy between men and women in Poland.

The content and conditions of participation in health programmes indicate a need for better recognition of this problem by local authorities.

Most health policy programmes focus on providing inhabitants with free vaccination and complementing limited access to healthcare (mainly in terms of rehabilitation). In general, lifestyle-related health risks are rarely considered and always in the context of a specific disease combined with screening or therapeutic activity.

The recruitment criteria for programmes are formal (age and diagnosed medical problem). They do not consider the recipients’ education level or health culture—their attitude towards their health, which is expressed by, among other things, practicing various harmful behaviours.

People affected with multiple risk factors, mostly men aged about 50 with vocational education, cannot expect effective support under health policy programmes proposed by local governments.

One can expect that both the lifestyle-related differences discussed in this article and their health outcomes will be exacerbated in the future. These are side effects of the COVID-19 pandemic, when harmful behaviours intensified, especially among already affected people. At the same time, the number of proposed health programmes has significantly decreased.

The results of this study should contribute to improving health programmes to reduce the prevalence of behavioural risk factors and their co-occurrence.

## Figures and Tables

**Table 1:** Characteristics of the analysed population and the four distinguished clusters within the scope of features included in the clustering procedure (number and percentage of people with a particular feature for qualitative variables; the median for age; limits of 95% confidence interval in square brackets; statistical significance of differences between clusters).

Characteristic	Total	Cluster, Size, % of the Total				Significance
		1—“The Youngest” 867 People 28.9% [27.3–30.5%]	2—“Multi-Risks” 789 People 26.3% [24.7–27.9%]	3—“The Oldest” 795 People 26.5% [24.9–28.1%]	4—“Healthy Lifestyle” 549 People 18.3% [16.9–19.7%]	
Percentage of women	1572 52.4% [50.6–54.2%]	471 54.3% [51.0–57.6%]	146 18.5% [15.8–21.2%]	524 65.9% [62.6–69.2%]	431 78.5% [75.1–81.9%]	*p* < 0.001 *
Age (median in years)	46 [45.2–46.8]	33 [31.9–34.1]	50 [48.8–51.2]	64 [62.7–65.3]	43 [41.3–44.7]	*p* < 0.001 *
Married/cohabitant	1965 65.5% [63.8–67.2%]	422 48.7% [45.3–52.0%]	698 88.5% [86.2–90.7%]	437 55.0% [51.5–58.4%]	408 74.3% [70.7–78.0%]	*p* < 0.001 (1–3) **
Tertiary education	524 17.5% [16.1–18.8%]	207 23.9% [21.0–26.7%]	66 8.4% [6.4–10.3%]	80 10.1% [8.0–12.2%]	171 31.1% [27.3–35.0%]	*p* < 0.001 (2–3) **
Secondary education	1262 42.1% [40.3–43.8%]	487 56.2% [52.9–59.5%]	238 30.2% [27.0–33.4%]	281 35.3% [32.0–38.7%]	256 46.6% [42.5–50.8%]	*p* < 0.001 (2–3) **
Basic vocational education	867 28.9% 27.3–30.5%]	138 15.9% [13.5–18.4%]	420 53.2% [49.8–56.7%]	225 28.3% [25.2–31.4%]	84 15.3% [12.3–18.3%]	*p* < 0.001 (1–4) **
Primary education	347 11.6% [10.4–12.7%]	35 4.0% [2.7–5.3%]	65 8.2% [6.3–10.2%]	209 26.3% [23.2–29.3%]	38 6.9% [4.8–9.0%]	*p* < 0.001 (1–4, 2–4) **
Living in a rural area	1184 39.5% [37.7–41.2%]	378 43.6% [40.3–46.9%]	337 42.7% [39.3–46.2%]	296 37.2% [33.9–40.6%]	173 31.5% [27.6–35.4%]	*p* < 0.001 (1–2, 2–3) **
Smoking	904 30.1% [28.5–31.8%]	255 29.4% [26.4–32.4%]	469 59.4% [56.0–62.9%]	67 8.4% [6.5–10.4%]	113 20.6% [17.2–24.0%]	*p* < 0.001 *
Alcohol problems	402 13.4% [12.2–14.6%]	82 9.5% [7.5–11.4%]	276 35.0% [31.7–38.3%]	15 1.9% [0.9–2.8%]	29 5.3% [3.4–7.2%]	*p* < 0.001 *
Lack of recreational physical activity	1398 46.6% [44.8–48.4%]	210 24.2% [21.4–27.1%]	502 63.6% [60.3–67.0%]	510 64.2% [60.8–67.5%]	176 32.1% [28.2–36.0%]	*p* < 0.001 (2–3) **
Overweight (BMI ≥ 25)	1489 49.6% [47.8–51.4%]	192 22.1% [19.4–24.9%]	575 72.9% [69.8–76.0%]	714 89.8% [87.7–91.9%]	8 1.5% [0.5–2.5%]	*p* < 0.001 *
Low fruit/vegetables in diet	2000 66.7% [65.0–68.4%]	523 60.3% [57.1–63.6%]	656 83.1% [80.5–85.8%]	542 68.2% [64.9–71.4%]	279 50.8% [46.6–55.0%]	*p* < 0.001 *
Low fish in diet	1772 59.1% [57.3–60.8%]	518 59.7% [56.5–63.0%]	602 76.3% [73.3–79.3%]	436 54.8% [51.4–58.3%]	216 39.3% [35.3–43.4%]	*p* < 0.001 (1–3) **
Unhealthy products in daily diet	2005 66.8% [65.1–68.4%]	623 71.9% [68.9–74.9%]	622 78.8% [76.0–81.7%]	439 55.2% [51.8–58.7%]	321 58.5% [54.3–62.6%]	*p* < 0.001 (3–4) **
Lack of medical prevention	1309 43.6% [41.9–45.4%]	639 73.7% [70.8–76.6%]	507 64.3% [60.9–67.6%]	162 20.4% [17.6–23.2%]	1 0.2% [0.0–0.5%]	*p* < 0.001 *

* Every cluster significantly differs from each other when the *p*-value is corrected for multiple comparisons. ** Every cluster significantly differs from each other except for pair (A,B) when the *p*-value is corrected for multiple comparisons (A,B—cluster numbers).

**Table 2:** Description of clusters identified according to the prevalence of behavioural health risk factors and socio-demographic characteristics in the Polish population.

Cluster	No. of People (% of the Total)	Median of Age [Years]	Description
1. “The youngest”	879 (29%)	33	The relative balance of sexes (54% of women); education over average (56% with secondary education, 24% with a university degree); least frequently married/cohabitant (49%); an average level of smoking (29%); drinking problems slightly less frequent than the average (9%); the highest physical activity (24% do not practice it for recreation); a relatively low prevalence of overweight (22%) and obesity (4%); average consumption of fish; consumption of vegetables and fruit slightly higher than average (40% eat 5 portions a day); often unhealthy diet—fast foods most frequently out of all clusters (23%—several times a week and 19%—once a week), sweets and sweet, carbonated beverages several times a week—59% and 55%, respectively; rarely undertake preventive health actions (examinations—24%, vaccination—5%).
2. “Multi- risks”	789 (26%)	50	Mainly male (81%); excess of people aged 40–59; the highest percentage of people with basic vocational education (55%), the lowest with a university degree (8%); the highest percentage of married/cohabitant (89%); very high percentage of smokers (59%) and people with drinking problems (35%); high share of people without recreational physical activity (64%); high prevalence of overweight (73%); the lowest consumption of vegetables, fruit, and fish, the highest of sweets, carbonated beverages and fast foods; preventive health actions less frequent than the average (medical examinations 34%, vaccination—6%).
3. “The oldest”	795 (27%)	64	Mostly women (66%); 62% aged 60+, 10% below 39 years of age; less educated (high excess of people with primary education—26%); the lowest percentage of smokers (8%); a very few drinking problems (2%); the least physically active; most frequently overweight (90%); average consumption of vegetables and fruit; consumption of fish slightly higher than average; the lowest consumption of unhealthy food; preventive actions more often than the average (80%).
4. “Healthy lifestyle”	549 (18%)	43	A great majority of women (79%); the highest education level—31% with a university degree, reduced primary (7%) and basic vocational education (15%); married or cohabitant more frequently than the average (74%); relatively low percentage of physically inactive people (32%); fewer smokers than the average (21%) and fewer often have alcohol problems (5%); low prevalence of overweight (1.5%) and only 1 case (0.2%) of obesity; the most frequent consumption of vegetables and fruit (recommended 5 portions a day—49%, only 2% of people do not eat vegetables and fruit daily) and fish (at least once a week—61%, 13%—several times a week); consumption of unhealthy food lower than the average (fast-food meals several times a week—8%, carbonated beverages—33%, sweets—51%); almost all take part in preventive medical examinations.

**Table 3:** Characteristics of the analysed population and four distinguished clusters within the scope of features not acknowledged in the clustering procedure (number and percentage of people with a particular feature; limits of 95% confidence interval in square brackets; statistical significance of differences between clusters).

Characteristic	Total	Cluster, Size, % of the Total				Significance
		1—“The Youngest” 867 People 28.9% [27.3–30.5%]	2—“Multi-Risks” 789 People 26.3% [24.7–27.9%]	3—“The Oldest” 795 People 26.5% [24.9–28.1%]	4—“Healthy Lifestyle” 549 People 18.3% [16.9–19.7%]	
Age up to 39 years	1130 37.7% [35.9–39.4%]	603 69.6% [66.5–72.6%]	223 28.3% [25.1–31.4%]	77 9.7% [7.6–11.7%]	227 41.3% [37.2–45.5%]	*p* < 0.001 *
Age of 60+	897 29.9% [28.3–31.5%]	77 8.9% [7.0–10.8%]	204 25.9% [22.8–28.9%]	493 62.0% [58.6–65.4%]	123 22.4% [18.9–25.9%]	*p* < 0.001 (2–4) **
Living in a city with population over 100,000	849 28.3% [26.7–29.9%]	198 22.8% [20.0–25.6%]	215 27.2% [24.1–30.4%]	236 29.7% [26.5–32.9%]	200 36.4% [32.4–40.5%]	*p* < 0.001 (1–2, 2–3, 3–4) **
Never smoked	1527 50.9% [49.1–52.7%]	505 58.2% [55.0–61.5%]	187 23.7% [20.7–26.7%]	497 62.5% [59.2–65.9%]	338 61.6% [57.5–65.6%]	*p* < 0.001 (1–3, 1–4, 3–4) **
Obesity (BMI ≥ 30)	337 11.2% [10.1–12.4%]	35 4.0% [2.7–5.3%]	112 14.2% [11.8–16.6%]	189 23.8% [20.8–26.7%]	1 0.2% [0–0.5%]	*p* < 0.001 *
Not eating vegetables and fruit daily	219 7.3% [6.4–8.2%]	62 7.2% [5.4–8.9%]	91 11.5% [9.3–13.8%]	55 6.9% [5.2–8.7%]	11 2.0% 0.8–3.2%]	*p* < 0.001 (1–3) **
Eating fast foods once a week or more often	823 27.4% [25.8–29.0%]	365 42.1% [38.8–45.4%]	255 32.3% [29.1–35.6%]	88 11.1% [8.9–13.3%]	115 20.9% [17.5–24.4%]	*p* < 0.001 *
Eating fast foods several times a week	417 13.9% [12.7–15.1%]	195 22.5% [19.7–25.3%]	133 16.9% [14.2–19.5%]	45 5.7% [4.1–7.3%]	44 8.0% [5.7–10.3%]	*p* < 0.001 (3–4) **
Taking carbonated drinks several times a week	1368 45.6% [43.8–47.4%]	476 54.9% [51.6–58.2%]	476 60.3% [56.9–63.7%]	236 29.7% [26.5–32.9%]	180 32.8% [28.9–36.7%]	*p* < 0.001 (1–2, 3–4) **
Eating sweets several times a week	1681 56.0% [54.3–57.8%]	508 58.6% [55.3–61.9%]	504 63.9% [60.5–67.2%]	389 48.9% [45.5–52.4%]	280 51.0% [46.8–55.2%]	*p* < 0.001 (1–2 3–4) **
Eating fish 2x/week or more often	239 8.0% [7.0–8.9%]	60 6.9% [5.2–8.6%]	28 3.5% [2.3–4.8%]	82 10.3% [8.2–12.4%]	69 12.6% [9.8–15.3%]	*p* < 0.001 (1–3, 3–4) **
Vaccination in the last 3 years	308 10.3% [9.2–11.4%]	46 5.3% [3.8–6.8%]	49 6.2% [4.5–7.9%]	106 13.3% [11.0–15.7%]	107 19.5% [16.2–22.8%]	*p* < 0.001 (1–2) **
Preventive medical examinations	1649 55.0% [53.2–56.7%]	205 23.6% [20.8–26.5%]	266 33.7% [30.4–37.0%]	630 79.2% [76.4–82.1%]	548 99.8% [99.5–100%]	*p* < 0.001 *
Did not need health care in the last year	862 28.7% [27.1–30.4%]	368 42.4% [39.2–45.7%]	267 33.8% [30.5–37.1%]	116 14.6% [12.1–17.0%]	111 20.2% [16.9–23.6%]	*p* < 0.001 *
Financial difficulties	595 19.8% [18.4–21.3%]	161 18.6% [16.0–21.2%]	182 23.1% [20.1–26.0%]	169 21.3% [18.4–24.1%]	83 15.1% [12.1–18.1%]	*p* = 0.002 (1–2, 1–3, 1–4, 2–3) **

* Every cluster significantly differs from each other when the *p*-value is corrected for multiple comparisons. ** Every cluster significantly differs from each other except for pair (A,B) when the *p*-value is corrected for multiple comparisons (A,B—cluster numbers).

**Table 4:** Health programmes that acknowledge intervention in the scope of several behavioural risk factors (BRF), submitted by Polish local governments for the assessment of AOTMiT in 2018.

No.	Title	BRF	Recipient Features; Age in Years
For patients			
1	Rehabilitation after gastrointestinal cancer	low physical activity + unhealthy diet	age of 18–64, after treatment of cancer
2	Rehabilitation of people with chronic diseases of the osteoarticular system	low physical activity + unhealthy diet	age of 18–64, with a chronic disease of the osteo-articular system
3	Rehabilitation of people with mental disorders caused by use of alcohol and other psychoactive substances	low physical activity + unhealthy diet + alcohol	age of 18–64, with mental disorders
4	Respiratory rehabilitation as a way back to professional and social activity	low physical activity + unhealthy diet + smoking	age of 18–64, with respiratory problems
5	Job-related diseases of the musculoskeletal system	low physical activity + unhealthy diet	professionally active at age of 18–65, with diseases of the musculoskeletal system
6	Rehabilitation of people with cardiovascular diseases	low physical activity + unhealthy diet + smoking	age 40+, with cardiovascular diseases
7	Nationwide lymphedema prevention programme after treatment of breast cancer	low physical activity + unhealthy diet	women, age of 45–64, after treatment of breast cancer
For people without a diagnosed disease			
8	Psychoeducational activity, prevention and early detection of dementia	low physical activity + unhealthy diet	age 55+
9	Prevention of civilisation diseases through early diagnostics and prevention of diabetes, overweight, and obesity	low physical activity + unhealthy diet	without limitations
10	Prevention of civilisation diseases through early diagnostics and prevention of diabetes, overweight, and obesity	low physical activity + unhealthy diet	age 16+
11	Prevention and detection of anxiety disorders	low physical activity + unhealthy diet + smoking	age of 18–64
12	Prevention and diagnosis of venous thromboembolism	low physical activity + unhealthy diet	age of 18–64
13	Primary prevention of cardiovascular disease	low physical activity + unhealthy diet + smoking	age of 30–60
14	Prevention and early detection of vascular diseases	low physical activity + unhealthy diet + smoking + alcohol	age of 35–66
15	Nutritional education and prevention of obesity-related diseases for children and adults	low physical activity + unhealthy diet + smoking	age 4+
16	Prevention and early detection of type 2 diabetes	low physical activity + unhealthy diet	age of 40–60
17	Early detection of overweightness and obesity among people of working age over 40	low physical activity + unhealthy diet	women 40–60, men 40–65
18	Prevention of obesity and type 2 diabetes	low physical activity + unhealthy diet	age 45+
19	Osteoporosis prevention	low physical activity + unhealthy diet + smoking + alcohol	age 55+/60+
20	Prevention and early detection of osteoporosis	unhealthy diet + smoking + alcohol	women 65+, men 70+

## Data Availability

The data that support the findings of this study are available from the corresponding author (AP) upon reasonable request.

## References

[1] Rechel B., Maresso A., Sagan A., Hernández-Quevedo C., Williams G., Richardson E., Jakubowski E., Nolte E. (2018). Organization and Financing of Public Health Services in Europe: Country Reports.

[2] OECD/European Union (2020). Health at a Glance: Europe 2020: State of Health in the EU Cycle.

[3] Institute for Health Metrics and Evaluation, University of Washington GBD Compare. https://vizhub.healthdata.org/gbd-compare/.

[4] European Commission, Eurostat Data Browser (2022). Life Expectancy by Age and Sex. https://ec.europa.eu/eurostat/databrowser/view/demo_mlexpec/default/table?lang=en.

[5] Wojtyniak B., Stokwiszewski J., Rabczenko D., Goryński P., Trochonowicz A., Madej T., Zdrojewski T., Wojtyniak B., Goryński P. (2022). Life expectancy and mortality of the population of Poland. Health Status of Polish Population and Its Determinants 2022.

[6] Poznańska A., Rabczenko D., Wojtyniak B., Wojtyniak B., Goryński P. (2022). Prevalence of behavioural health risk factors and its changes during the COVID-19 pandemic. Health Status of Polish Population and Its Determinants 2022.

[7] Buck D., Frosini F. (2012). Clustering of Unhealthy Behaviours over Time: Implications for Policy and Practice.

[8] Schuit A.J., van Loon A.J., Tijhuis M., Ocké M. (2002). Clustering of lifestyle risk factors in a general adult population. Prev. Med..

[9] Noble N., Paul C., Turon H., Oldmeadow C. (2015). Which modifiable health risk behaviours are related? A systematic review of the clustering of Smoking, Nutrition, Alcohol and Physical activity (‘SNAP’) health risk factors. Prev. Med..

[10] Meader N., King K., Moe-Byrne T. (2016). A systematic review on the clustering and co-occurrence of multiple risk behaviours. BMC Public Health.

[11] Birch J., Petty R., Hooper L., Bauld L., Rosenberg G., Vohra J. (2019). Clustering of behavioural risk factors for health in UK adults in 2016: A cross-sectional survey. J. Public Health.

[12] Finger J.D., Hoebel J., Kuntz B., Kuhnert R., Zeiher J., Mensink G.B., Lampert T. (2019). Educational differences in the prevalence of behavioural risk factors in Germany and the EU–Results from the European Health Interview Survey (EHIS) 2. J. Health Monit..

[13] Conry M.C., Morgan K., Curry P., McGee H., Harrington J., Ward M., Shelley E. (2011). The clustering of health behaviours in Ireland and their relationship with mental health, self-rated health and quality of life. BMC Public Health.

[14] Poortinga W. (2007). The prevalence and clustering of four major lifestyle risk factors in an English adult population. Prev. Med..

[15] Kaczynski A.T., Manske S.R., Mannell R.C., Grewal K. (2008). Smoking and physical activity: A systematic review. Am. J. Health Behav..

[16] Kaleta D., Makowiec-Dabrowska T., Polańska K., Dziankowska-Zaborszczyk E., Drygas W. (2009). Tobacco smoking and other negative lifestyle behaviors among economically active individuals. Med. Pract..

[17] Kwaśniewska M., Pikala M., Aranowska A., Bielecki W., Kozakiewicz K., Pająk A., Tykarski A., Zdrojewski T., Nadrowski P., Piwoński J. (2021). Ten-year changes in adherence to a healthy lifestyle: The results of the WOBASZ surveys. Pol. Arch. Intern. Med..

[18] National Institute of Public Health NIH—National Research Institute, ProfiBaza. (In Polish). https://profibaza.pzh.gov.pl/AOTMIT.

[19] Schulz D.N., Kremers S.P., Vandelanotte C., Van Adrichem M.J., Schneider F., Candel M.J., de Vries H. (2014). Effects of a web-based tailored multiple-lifestyle intervention for adults: A two-year randomized controlled trial comparing sequential and simultaneous delivery modes. J. Med. Internet Res..

[20] Meader N., King K., Wright K., Graham H.M., Petticrew M., Power C., White M., Sowden A.J. (2017). Multiple Risk Behavior Interventions: Meta-analyses of RCTs. Am. J. Prev. Med..

[21] Betsch C., Böhm R., Airhihenbuwa C.O., Butler R., Chapman G.B., Haase N., Herrmann B., Igarashi T., Kitayama S., Korn L. (2016). Improving Medical Decision Making and Health Promotion through Culture-Sensitive Health Communication: An Agenda for Science and Practice. Med. Decis. Mak..

[22] Krebs P., Prochaska J.O., Rossi J.S. (2010). A meta-analysis of computer-tailored interventions for health behavior change. Prev. Med..

[23] Bol N., Smit E.S., Lustria M.L.A. (2020). Tailored health communication: Opportunities and challenges in the digital era. Digit Health.

[24] Crane M.M., Halloway S., Walts Z.L., Gavin K.L., Moss A., Westrick J.C., Appelhans B.M. (2021). Behavioural interventions for CVD risk reduction for blue-collar workers: A systematic review. J. Epidemiol. Community Health.

[25] Zabaleta-del-Olmo E., Pombo H., Pons-Vigués M., Casajuana-Closas M., Pujol-Ribera E., López-Jiménez T., Cabezas-Peña C., Martín-Borràs C., Serrano-Blanco A., Rubio-Valera M. (2018). Complex multiple risk intervention to promote healthy behaviours in people between 45 to 75 years attended in primary health care (EIRA study): Study protocol for a hybrid trial. BMC Public Health.

[26] Wolfenden L., Goldman S., Stacey F.G., Grady A., Kingsland M., Williams C.M., Wiggers J., Milat A., Rissel C., Bauman A. (2018). Strategies to improve the implementation of workplace-based policies or practices targeting tobacco, alcohol, diet, physical activity and obesity. Cochrane Database Syst. Rev..

[27] Thomas R.E., Baker P.R.A., Thomas B.C., Lorenzetti D.L. (2015). Family-based programmes for preventing smoking by children and adolescents. Cochrane Database Syst. Rev..

[28] Power C., Parsons T. (2000). Nutritional and other influences in childhood as predictors of adult. Proc. Nutr. Soc..

[29] Mazur J., Małkowska-Szkutnik A. (2018). School-Age Children’s Health in 2018 According to the New HBSC 2018 Survey Model.

[30] Kriaucioniene V., Bagdonaviciene L., Rodríguez-Pérez C., Petkeviciene J. (2020). Associations between Changes in Health Behaviours and Body Weight during the COVID-19 Quarantine in Lithuania: The Lithuanian COVIDiet Study. Nutrients.

[31] Ferrante G., Camussi E., Piccinelli C., Senore C., Armaroli P., Ortale A., Garena F., Giordano L. (2020). Did social isolation during the SARS-CoV-2 epidemic have an impact on the lifestyles of citizens?. Epidemiol. Prev..

[32] Di Renzo L., Gualtieri P., Pivari F., Soldati L., Attinà A., Cinelli G., Leggeri C., Caparello G., Barrea L., Scerbo F. (2020). Eating habits and lifestyle changes during COVID-19 lockdown: An Italian survey. J. Transl. Med..

[33] McBride E., Arden M.A., Chater A., Chilcot J. (2021). The impact of COVID-19 on health behaviour, well-being, and long-term physical health. Br. J. Health Psychol..

[34] Sidor A., Rzymski P. (2020). Dietary Choices and Habits during COVID-19 Lockdown: Experience from Poland. Nutrients.

